# The ecological controls on the prevalence of candidate division TM7 in polar regions

**DOI:** 10.3389/fmicb.2014.00345

**Published:** 2014-07-15

**Authors:** Tristrom J. Winsley, Ian Snape, John McKinlay, Jonny Stark, Josie M. van Dorst, Mukan Ji, Belinda C. Ferrari, Steven D. Siciliano

**Affiliations:** ^1^Australian Antarctic Division, Department of the EnvironmentKingston, TAS, Australia; ^2^Department of Soil Science, University of SaskatchewanSaskatoon, SK, Canada; ^3^Faculty of Science, School of Biotechnology and Biomolecular Sciences, University of New South WalesKensington, NSW, Australia

**Keywords:** candidate pylum TM7, soil bacteria, Arctic, Antarctic, siderophores, bacterial culturing

## Abstract

The candidate division TM7 is ubiquitous and yet uncultured phylum of the Bacteria that encompasses a commonly environmental associated clade, TM7-1, and a “host-associated” clade, TM7-3. However, as members of the TM7 phylum have not been cultured, little is known about what differs between these two clades. We hypothesized that these clades would have different environmental niches. To test this, we used a large-scale global soil dataset, encompassing 223 soil samples, their environmental parameters and associated bacterial 16S rRNA gene sequence data. We correlated chemical, physical and biological parameters of each soil with the relative abundance of the two major classes of the phylum to deduce factors that influence the groups' seemingly ubiquitous nature. The two classes of the phylum (TM7-1 and TM7-3) were indeed distinct from each other in their habitat requirements. A key determinant of each class' prevalence appears to be the pH of the soil. The class TM7-1 displays a facultative anaerobic nature with correlations to more acidic soils with total iron, silicon, titanium and copper indicating a potential for siderophore production. However, the TM7-3 class shows a more classical oligotrophic, heterotroph nature with a preference for more alkaline soils, and a probable pathogenic role with correlations to extractable iron, sodium and phosphate. In addition, the TM7-3 was abundant in diesel contaminated soils highlighting a resilient nature along with a possible carbon source. In addition to this both classes had unique co-occurrence relationships with other bacterial phyla. In particular, both groups had opposing correlations to the Gemmatimonadetes phylum, with the TM7-3 class seemingly being outcompeted by this phylum to result in a negative correlation. These ecological controls allow the characteristics of a TM7 phylum preferred niche to be defined and give insight into possible avenues for cultivation of this previously uncultured group.

## Introduction

The candidate bacterial division TM7 is highly ubiquitous and has been observed in environments ranging from soils, sediments and wastewater sludge and has also be associated with human diseases such as periodontitis and inflammatory bowel disease (IBD) (Hugenholtz et al., 2001; Dinis et al., 2011). Hugenholtz et al. (2001) pioneered the investigation of the phylum after it was first discovered in a peat bog (Rheims et al., 1996; Hugenholtz et al., 2001). They developed and employed probes and primers to target the bacteria *in situ* and these were subsequently used in successive investigations by other researchers (Ouverney et al., 2003; Ferrari et al., 2005; Podar et al., 2007; Dinis et al., 2011). A brief and relatively undescribed cultivation success was obtained with a microcolony of TM7 on R2A when micromanipulated from sludge (Hugenholtz, 2002). This suggests that while the bacteria have not yet been isolated in pure culture they are, in fact, not “unculturable” and that additional environmental information would help define the “niche” in which these bacteria grow. Of the many sites where the TM7 phylum has been detected, two particular environments have generated significant interest: wastewater treatment plants and human associated TM7 (Brinig et al., 2003; Xia et al., 2007; Kuehbacher et al., 2008; Honda and Littman, 2012; Mielczarek et al., 2012). If more information can be obtained about members of the phylum, particularly through the study of pure cultures, prospects such as stimulation of wastewater treatment processes and discovery of drug targets for clinical conditions involving TM7 would become possible.

Within the phylum, two major subdivisions exist, representing approximately class-level clades (Dinis et al., 2011). These clades (TM7-1 and TM7-3 as they are currently labeled in the GreenGenes repository) are commonly associated with particular sources of TM7 members (McDonald et al., 2012). Dinis et al. (2011) purported that TM7-1 are most often affiliated with “environmental” samples such as soils and sediment, whereas TM7-3 member species are identified in samples of “animal” origin. The extent of knowledge on these two classes is limited but with recent genome sequencing efforts by Albertsen et al. (2013), the complete genome has become available for 4 members of the TM7-1 class recovered from wastewater. This has highlighted the capacity for these members to utilize simple sugars leading to a proposed name of “*Saccharibacteria”* for the phylum. However, this advance represents a limited diversity, with only 4 similar members of the TM7-1 class being sequenced and leaves a gap in knowledge regarding the TM7-3 class as well as many members of the TM7-1 class. For comprehensive understanding of the phylum not only do more species from the TM7 division need to be sequenced but examined *in situ* with the goal of obtaining cultures to study.

To uncover the functional and nutritional requirements of the candidate division TM7 we investigated the environmental (chemical and geographic parameters) and biological factors that correlate with the abundance of bacteria from the TM7. This information will contribute to cultivation efforts for a group of bacteria that is highly ubiquitous yet has so far not been cultured *in vitro.*

## Materials and methods

### Sample collection

Two hundred and twenty five soil samples spanning the Arctic and Antarctica were collected in a geospatial transect design, from 8 locations as previously described (Table S1) (Palmer et al., in press). Transects were designed to give high levels of fine- and wide-scale replication, with 3 parallel transects running 300 m across a selected site at each location. For each sample, geographic data was also collected in the form of latitude and longitude, elevation, aspect (relation of the majority of the face of the sampling area to the globe, represented as a number from 0 to 360°) and slope (angle of the sampling area from the horizontal). These last two factors bear significance to water runoff and sunlight incidence respectively. One of the locations, Casey station, has a legacy of diesel fuels spills. A number of the samples from this location contain high levels of contamination with a special Antarctic blend of diesel fuel (Total Petroleum Hydrocarbons (TPH) C9-C40 up to 22,000 mg fuel kg^−1^ soil). At each sampling site, surface debris (stones etc.) were removed and 100 g of soil was collected from a few cm below the surface. One single collection (i.e., 223 total samples) was made at each sampling site which was tagged and stored at −80 for transport back to Australia for analysis.

### DNA extraction and sequencing

The soil samples were homogenized and a portion (300 mg) was weighed out for mechanical extraction of metagenomic DNA. This was achieved using a FastDNA SPIN kit for soils (MP Biomedicals, Seven Hills, NSW, Australia), according to the manufacturers protocol. Extracts were quantified with picogreen (Invitrogen, Mount Waverley, VIC, Australia). One hundred ng of each sample were sent to a sequencing facility in Lubbock, Texas (Research and Testing Lab, Lubbock, TX, USA) for 16S rRNA gene amplicon sequencing using the PCR primers 27F and 519R (Lane, 1991) on the 454 FLX titanium platform as described by Dowd et al. (2008) (Roche Life Sciences, Branford, CT, USA). These primers were selected to provide a longer amplicon than the commonly used V4 and V6 region primers as the 454 platform is capable of producing fragments up to 500 bp.

### Sequence data processing

The sequence data returned from the Research and Testing lab was processed in a pipeline that predominantly implemented the mothur software package as previously described (Schloss et al., 2009; Siciliano et al., in press). Briefly, this involved denoising, dereplication and quality screening of the reads (Palmer et al., in press). Subsequently, the reads were aligned using the NAST algorithm to the SILVA seed database and chimaeric artifacts were removed before pre-clustering at 1% dissimilarity to alleviate the per-base error rate of the sequencing platform. “Clean” reads were then clustered into operational taxonomic units (OTU) at a 96% similarity which is determined to be approximately species-level for the V1–V3 region as compared to the 97% threshold for full-length 16S sequences (Kim et al., 2011). The OTUs were then taxonomically identified using the naïve Bayesian classifier in mother, searching their nucleotide sequence against a curated version of the GreenGenes database (McDonald et al., 2012; Werner et al., 2012) to accommodate only the V1-V3 region with a search cut-off of 80% confidence for short reads (Wang and Qian, 2009). The samples were then subsampled to a number of reads (1955) that represented the lowest sample to provide unbiased comparison across samples. Estimates of bacterial diversity were generated from OTU-by-sample matrices providing species richness, and evenness values. For the TM7 OTUs, UniFrac analysis was performed (Lozupone et al., 2011); this provided a dissimilarity matrix across all sites based solely upon the TM7 population. Additionally, the TM7 OTUs were assigned a class-level taxonomic rank within the OTU-by-sample matrix to allow designation of the different classes within the phylum. These class abundances were represented as relative to the entire microbial community of each sample, not as a percentage of the TM7 phylum as a whole. This included the two classes TM7-1 and TM7-3, as well as those OTUs that cannot be classified into one of these two classes due to the paucity of comparable reference sequences (TM7-unc). The unclassified TM7 sequences were not included in many of the analyses since their taxonomy was uncertain.

### Chemical analysis

Chemical properties of each soil were analyzed by various methods as previously described and are listed in Table S2 (Palmer et al., in press). Briefly these were done by chromatic methods for extractable chemicals, X-ray fluorescence, Kjeldahl digestion, combustion and NDIR gas analysis (carbon only) and ICP-OES of the Melich-3 extraction from the soils to detect the exchangeable cation fractions. Conductivity, pH and grain size [mud (<63 μm—encompassing clay and silt), sand (63–2000 μm), gravel (>2 mm)] were also measured.

### Phylogenetic analysis

**Figure 1:**
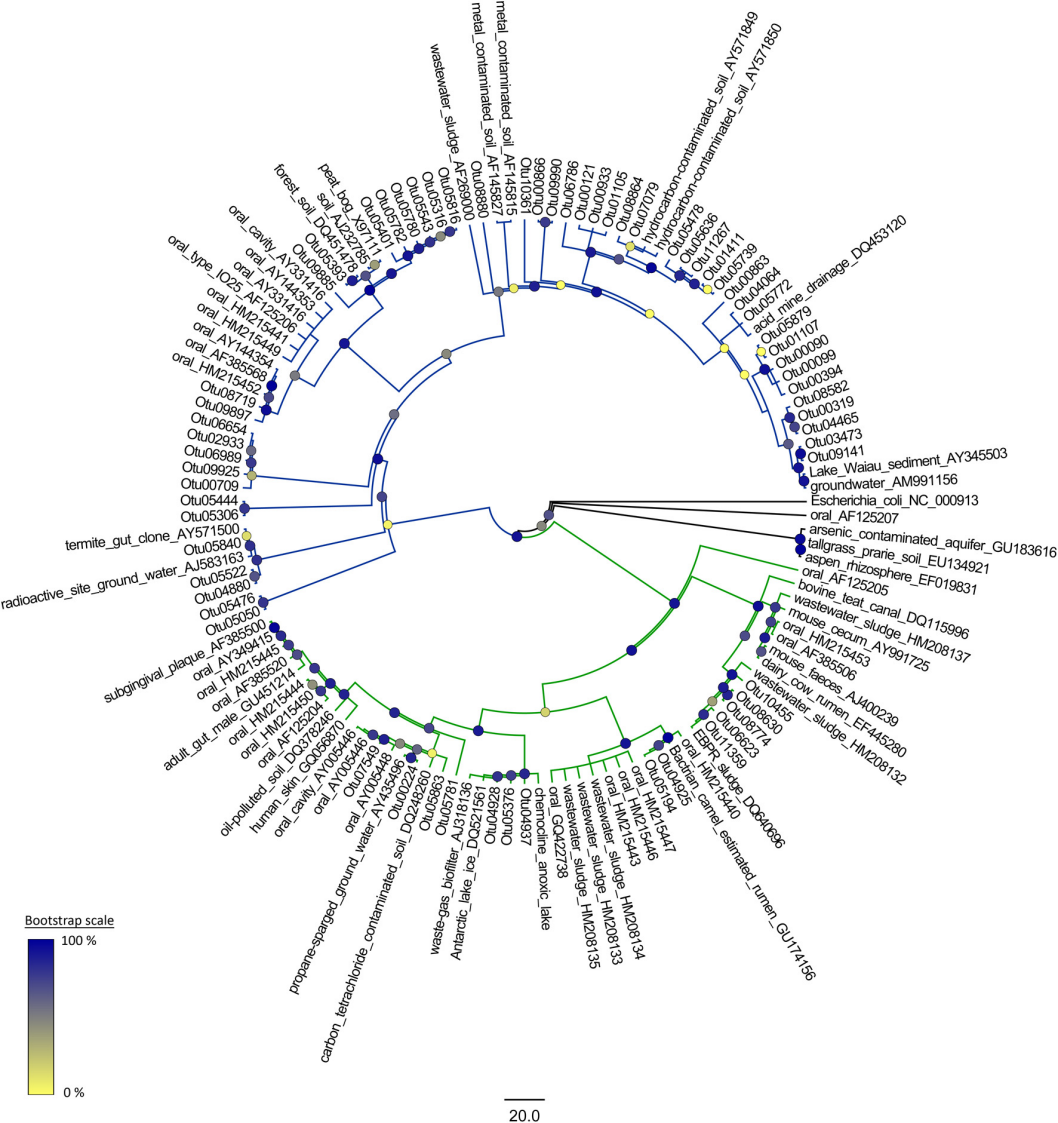
**A phylogenetic reconstruction of the candidate division TM7**. The reference sequences are obtained from GreenGenes. The tree is a maximum-likelihood phylogeny generated in FastTree 2 with a general time reversible model and 1000 bootstrap replications. TM7-1 and TM7-3 are shown with green and blue branches respectively.

Representative sequences from each species-level OTU were obtained using the mothur software package. These sequences were used to construct a phylogeny of the TM7 phylum along with reference sequences of bacteria from the TM7 phylum obtained from GreenGenes. These reference sequences were selected to cover a range of environments where TM7 bacteria have been detected including clinical, industrial and contaminated environments. A reference sequence for *Escherichia coli* was used as an outgroup. A maximum-likelihood tree was constructed using the FastTree 2 program. This involved using the NAST algorithm in mothur (Desantis et al., 2006; Schloss et al., 2009) to align the TM7 OTUs and the reference sequences and then generating a neighbor-joining tree. Refinement of this was achieved by using a combination of subtree-prune-regraft (SPR) and nearest-neighbor interchanges (NNI). A temporary minimum evolution tree was generated which was then optimized using a general-time-reversible model to produce a maximum-likelihood tree. The topology of the tree was tested with 1000 bootstrap replicates (Figure 1) (Price et al., 2010).

### Correlations of TM7 class abundances with soil characteristics

Biological data were converted into an OTU abundance-by-sample matrix to allow correlation of the various bacterial phyla detected in the dataset with the TM7 phylum. In addition to this the environmental data (chemical and geographical) were inspected to determine normality of the distribution of the data and those that required transformation were manipulated to reduce the skewness and kurtosis by 1 of 4 methods (Table S2). The curated dataset was imported in the R software package (R Development Core Team, 2010) and correlations were examined between each TM7 class and the various biological and environmental parameters. This was achieved by plotting the data, fitting a linear model to the plot, and subsequently calculating the correlation coefficient with its significance. Correlations were represented visually by using a “holosphere” where significant relationships are plotted on a radial axis plot (**Figure 3**).

### Multivariate analysis

Multivariate analysis was conducted using Primer + Permanova 6 (Clarke, 1993). To explore the relationship between samples based on the presence of TM7, Bray-Curtis dissimilarities (Bray and Curtis, 1957) were subjected to unconstrained ordination using Kruskal's (1964) non-metric multidimensional scaling (nMDS) (Kruskal, 1964). An optimal nMDS configuration was achieved by choosing the lowest stress score from 50 random starts (Figure S1). The TM7 data were also ordinated using distance-based redundancy analysis (dbRDA), a method for assessing the degree to which variability in a community dissimilarity matrix can be explained by a matrix of environmental covariates (termed constraints). This was performed with a UniFrac dissimilarity matrix as opposed to OTU abundance data. The correlations of the soil parameters with the axes of the dbRDA were overlayed on top of the ordination and only those with significant correlations were displayed. In addition to the vector overlay of predictor variables, the abundance of each class of TM7 bacteria was added via bubble plotting (**Figure 3**). In addition to this, distance-based linear modeling (DistLM) was performed in Primer to rank the influence of the variables with significant correlations on the TM7 phylum (Table S4).

### Network co-occurrence analysis

The OTU-by-sample matrix was imported into PRIMER software and aggregated into phyla with the taxonomy derived previously except for the TM7 phylum which was grouped by class. These data were tabulated and using the SparrCC algorithm in the mothur software package co-occurrence analysis was performed (Schloss et al., 2009; Friedman and Alm, 2012). Output files from the SparrCC analysis were visualized in Cytoscape (V3.1.0, Windows version). The network was analyzed considering only the TM7 subset of nodes and the edges were colored blue for a positive relationship and red for a negative one (**Figure 4**).

## Results

### Diversity and distribution of TM7 bacteria within polar soils

Gene sequences for bacteria belonging to the TM7 phylum were detected in approximately half of the samples interrogated 48.33% (Figure S1). The relative abundance of the phylum with the samples where TM7 bacteria were present ranged from 0.05 to 2.23% of the microbial community. Among the TM7 OTUs detected there were representatives of both of the classes in each pole though the majority of TM7-3 species OTUs were from the Antarctic continent while TM7-1 class OTUs were observed almost equally among the poles. Out of the 76 OTUs, the majority were detected exclusively in the North or South poles, though a small number (7) were more cosmopolitan occurring in both hemispheres.

Of the 76 different species-level OTUs belonging to the TM7 phylum found in the dataset, 11 of these OTUs were present in more than 10 samples indicating some dominant TM7 OTUs within the polar soils. The majority of the TM7-3 class OTUs were similar to those species identified in clinical samples, while a significant number of the TM7-1 OTU sequences bore homology to representatives from environmental samples often with a contamination source (Figure 1).

### Distance-based redundancy analysis and linear modeling

**Figure 2:**
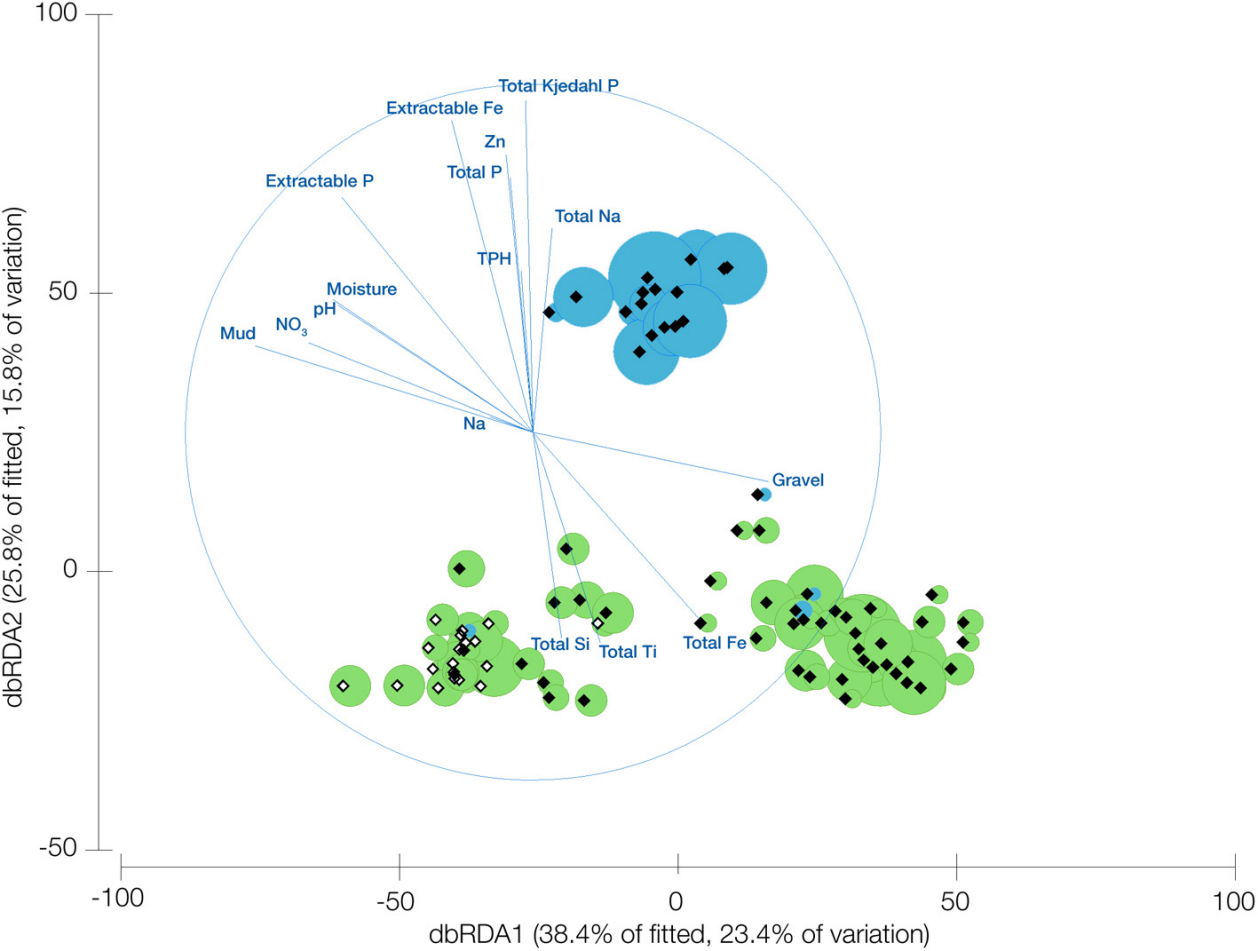
**Distance based redundancy analysis of UniFrac dissimilarity scores for samples where bacteria from the candidate division TM7 are present**. Overlayed are vectors representing the correlation of edaphic predictor variables and bubbles representing the relative abundance of the different TM7 classes (TM7-1 green, TM7-3 blue). Open diamonds represent Arctic samples and shaded represent Antarctic samples.

Ordination of the TM7 OTUs from the UniFrac matrix revealed that the two classes of TM7 examined have significantly different environmental requirements (Figure 2) with the first two axes explaining 39.2% of the variation. It was apparent that the samples cluster into three distinct groups, two dominated by OTUs from TM7-1 and the other by TM7-3. The vector correlations of the edaphic parameters were distinctly divided between the clusters containing TM7-1 and the cluster with TM7-3. Positive correlations with high total iron (expressed as Fe_2_O_3_), silicon, titanium and copper as well as gravel content all seem to be driving the prevalence of TM7-1, along with correlations to low pH and increased moisture. On the other hand, the TM7-3 class has positive correlations with high phosphorus, extractable iron, and various forms of sodium. They also favor a more alkaline soil with less moisture and finer soil particles than TM7-1.

Linear modeling of the variables determined to be significantly influential on the TM7 phylum (DistLM analysis) showed that the most important variable is phosphorus followed by TPH and then pH. Mineral properties of the soils comprised the next most important factors with textural properties least important to the phylum's presence in a soil (Table S4).

### Univariate correlation of chemical, biological and geographic parameters of the soils with the TM7 phylum

**Figure 3:**
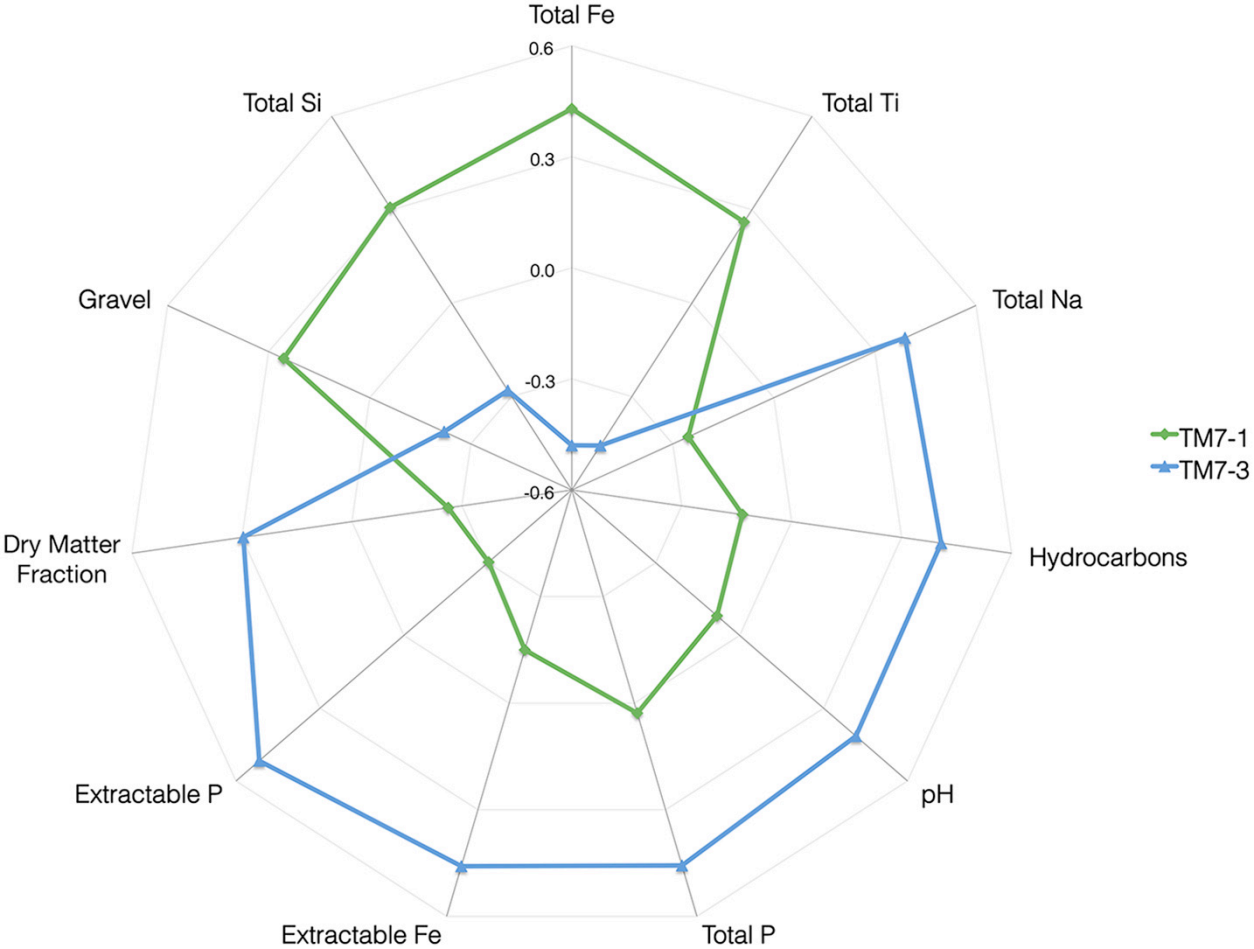
**A “Holosphere” representation of correlations of the abundance of the two classes of TM7 with various soil properties**. The scale on the axis incorporates both positive (+0.6) and negative (−0.6) correlations.

Analysis of the measured chemical, biological (richness and evenness) as well as geographic parameters revealed significant correlations between the relative abundance of the different TM7 classes and the predictor variables (Table S2). This revealed a similar correlation profile to the dbRDA and confirmed the relationships that were previously observed. The “holosphere” chart illustrates the correlations observed, highlighting the distinct difference between the two classes of the TM7 phylum (Figure 3).

Interestingly, only TM7-3 showed a correlation to the biological parameters of the soils, notably negative correlations with bacterial richness and evenness (Table S3). The single most important site-specific and geographic factor in the distribution of bacteria from the TM7 phylum within polar soils is the impact of human activity. Casey station, one of the locations selected for sampling in this study, is a long-standing research station in Antarctica that has had a number of fuel spills and significant human and vehicular activity. This apparently provided the necessary conditions for bacteria from the class TM7-3 to dominate to the complete exclusion of TM7-1.

### Co-occurrence analysis of phyla associated with TM7

**Figure 4:**
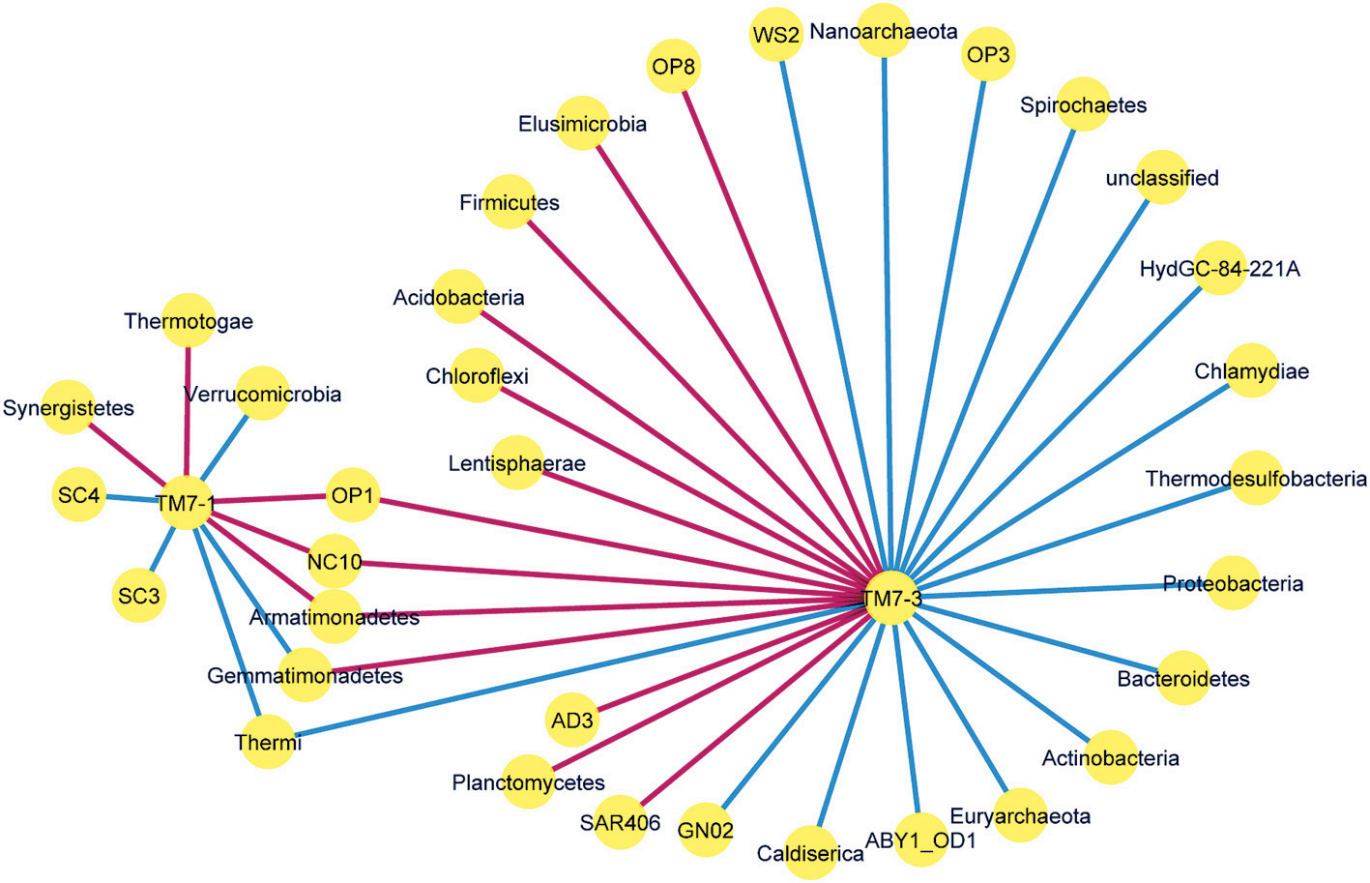
**Network co-occurrence analysis of the TM7 classes, TM7-1 and TM7-3 with respect to bacterial phyla present in the polar soil dataset**. Only significant relationships are shown. Edges are blue if the relationship between nodes is positive and red if negative.

Analysis of the phyla co-occurring with the two classes of the TM7 phylum showed several significant (*p* < 0.05) associations. The patterns of phyla associated with TM7 classes contained a number of unique associations for each class and 5 phyla that were shared (Figure 4). Of these, the *Gemmatimodetes* showed a differing direction of correlation with the TM7 classes, positively correlated with TM7-1 and negatively with TM7-3. The remaining shared phyla bore similar directional correlations with the two classes. For the TM7-1 class, the division between positive and negative associations was even; for the TM7-3 class, 55% of the associations were positive and 45% were negative.

### Characteristics of a niche favoring TM7 bacteria

The two classes of TM7 display clearly different requirements. TM7-1 favor soils that are acidic with an abundance of total iron as well as silicon, titanium and copper. Additionally, a wetter soil with a larger grain size was also associated with greater numbers of TM7-1. However, members of the TM7-3 class have a correlation with soils with high levels of total and extractable phosphorus, high levels of extractable iron, sodium, zinc, and aluminum. Interestingly, they thrived at the site where there were hydrocarbon (C9-C40) fuel spills. The sites TM7-3 favored tended to be alkaline, with finer grain size and less moisture than TM7-1. We also explored sites were no TM7 16S sequences were detected and there was no discernible pattern, chemical or biological that we could detect.

## Discussion

This study aimed to determine the ecological controls, both physical and biological, that affect the prevalence of bacteria from the candidate division TM7. With a dataset that contained 223 samples, including 13 with TPH contamination, we were able to identify a suite of conditions that likely provide a favorable milieu for TM7 bacteria. With relative abundances ranging from 0.05% up to 2.23% (approximately a 45 fold increase) it is clear that some conditions are much more conducive to the growth of TM7. These conditions were different for the two classes of the TM7 phylum and we were able to suggest the environmental conditions that would be potentially preferred by each group.

When analysing the phylogeny of the OTUs that we detected, there was an even distribution among the two classes of TM7 (as well as some unclassified OTUs) despite the fact that all the sequences were obtained from soil. This presence of a significant number of members of the TM7-3 class varies from observations by Dinis et al. (2011) who suggested that subdivision 1 (TM7-1) consisted almost exclusively of OTUs identified in environmental samples, while subdivision 2 (TM7-3) was mostly OTUs from clinical and animal sources (Dinis et al., 2011). However, there are some exceptions to this rule within their data; many wastewater clones were detected in both clades as well as numerous soil and contaminated site clones. It is possible that, provided the right conditions are met, an OTU could exist in both clinical and environmental scenarios. This is evidenced by the IO25 phylotype of the TM7 phylum having been observed in periodontitis cases and soils (Ouverney et al., 2003; Dinis et al., 2011). Another potential explanation for TM7-3 in soils is that most of the TM7-3 OTUs were detected around Casey station, a center of human activity. It is possible that the source of TM7-3 within these soils is human in origin.

Based on the phylogenetic reconstruction of the TM7 phylum, the observation that members of both classes can be found in similar environments is likely influenced by the pH of the soil. It is probable that members of the TM7-1 class and TM7-3 class are present in all the samples but the soil parent material, is at a particular pH and so one of the two classes will thrive. As the pH of the soils measured here varied from approximately 5 to 8, and in other environments, such as the periodontal pocket, can vary from around 5 to 9 (Galgut, 2001), it would suggest that pH is a very significant control on the presence of TM7 OTUs in an environment. Therefore, it is likely that when the pH in an environment, such as soil or the human mouth, tends toward an acidic level, the conditions for the TM7-1 class are met and this clade can establish colonies. The converse is true for the TM7-3 class where an alkaline environment will possibly provide suitable conditions for growth. The influence of pH on the different TM7 clades is probably the single biggest controlling factor for the abundance of each group. This holds true with previous observations of pH controlling microbial community composition (Siciliano et al., in press). Several other research groups have also identified pH as a key factor in controlling which species prevail in soil microbial communities (Fierer and Jackson, 2006; Lauber et al., 2009; Chu et al., 2010). In addition to pH, the moisture of the soils was seen to be a factor dividing the two groups. The correlation with moisture and larger grain size of TM7-1 suggests that the group may use alternative electron acceptors under oxygen-limited conditions. This would likely be due to the anoxic conditions provided by higher saturation in finer soils forcing out the available oxygen.

While pH plays an important role in the determination of which members of the TM7 phylum are present, it is not the most significant factor in providing conditions for the group. DistLM analsysi showed phosphorus content to be the critical variable in providing an environment for these bacteria. This was followed by TPH as an influential factor, though as TPH was only present in a small number of sites and has such a dramatic effect on the microbial community, it is difficult to deduce whether it has a direct influence on TM7. After phosphorus, TPH, and pH, then mineralogy appears as an important component of this phylum's requirements followed by the microphysical property of the soil (grainsize, moisture). Phosphorus has often been considered an important soil property and has a strong association with microbial activity (Jonasson et al., 1996). It is therefore not surprising that it appear to influence the presence of TM7 bacteria. The P levels within the polar soils analyzed range highly, and in some cases exceed levels observed in wastewater treatment, which has been noted to have soluble phosphate concentration around 18 mg/L (Bond et al., 1995). The mineral component of a soil has also been shown to be key in controlling which bacteria inhabit a soil (Carson et al., 2009). Textural properties of a soil affect many other parameters and so can have an indirect effect as well as a direct contribution to the proliferation of a bacterial species. The link between texture and pH could be responsible for the effect observed in this study (Etter and Grassle, 1992).

When considering the two classes separately, it becomes apparent that TM7 bacteria are likely to play different roles in various environments. TM7-1 bacteria display correlations with acidic, coarse-grained, iron and titanium rich siliceous sediments. The *Pseudomonads*, a group of the *Proteobacteria*, produce pyoverdine siderophores to chelate iron and titanium from the environment. Additionally, Gram-positive bacteria such as *Bacillus* spp. along with the *Pseudomonads* have a unique interaction with SiO_2_, which creates a net negative charge within the cell and is linked to the production of pyoverdine siderophores (Gordienko and Kurdish, 2005, 2007; Chobotar'ov et al., 2010). Since TM7 are Gram-positive and correlate with these three metals as well as a low pH (siderophores act optimally in acidic conditions and are required when iron is mostly in the insoluble Fe(III) form), it could be that the TM7-1 class is capable of producing pyoverdine-like siderophores. While the measure we obtained was of total silicon in the parent material, the sites we sampled that were high in silicon, were also low in magnesium and iron, reflecting a felsic bedrock substrate (high-grade felsic gneisses) (Marshak, 2001). In combination with a preference to larger grain sizes it is possible that these bacteria from TM7-1 might be adhering to metal oxide particles which can then provide a substrate for the formation of biofilms and successful growth of colonies.

The TM7-3 class had three major correlations, extractable iron and various forms of phosphorus and sodium. In addition to this, the class displayed an association with drier soils of a higher pH with a finer grain size. An affinity for available iron and sodium is often linked with pathogens and may explain the presence of TM7 bacteria disease scenarios (Hase et al., 2001; Gordienko and Kurdish, 2005; Bonder et al., 2012). However, correlations with various forms of phosphorus, may provide a clue to cultivation efforts. This is of significance as TM7 bacteria are often detected in wastewater treatment plants where enhanced biological phosphate removal occurs (Bond et al., 1995; Hugenholtz et al., 2001; Kong et al., 2007), indicating that they are not only tolerant of high phosphorus levels but potentially require elevated levels as essential nutrients. In addition to these correlations, the TM7-3 class was abundant whenever there was a presence of hydrocarbon contamination at a site. Two studies have identified hydrocarbon degradation (benzene and toluene) by members of the TM7 phylum through the use of stable isotope probing (SIP) (Luo et al., 2009; Xie et al., 2011). Therefore, either the hydrocarbon fuel contaminating the soil at Casey station is merely tolerated by TM7-3 bacteria or it may be a substrate for the TM7 bacteria contributing to increased abundance. Albertsen et al. (2013) in their efforts to sequence the genome of members of the TM7 phylum did not identify any genes that encode hydrocarbon degradation though Podar et al. (2007), were able to identify several genes relating to the management of toxic compounds; these included 5 H+ antiporters (from the drug H+antiporter 1 family) that are involved in drug resistance, lipopolysaccharide exporters from the ABC transporter family) that export lipid A, heavy metals and macrolide antibiotics. They also identified the cytochrome p450 gene that is commonly associated with resistance to toxic compounds. These factors suggest that the members of the TM7 phylum are resilient to harsh environments, likely contributing to their ubiquitous nature.

The correlations between ecological factors and TM7 species abundance observed in this study provide evidence for a “preferred” niche of bacteria from the TM7 phylum within polar soils. The two classes of the phylum (TM7-1 and TM7-3) are distinct from each other in their requirements. A key determinant of each class' prevalence appears to be the pH of the environment. Whether this is a correlate of the attributes of the soil parent material and is coincidental requires experimental validation to resolve. In addition further empirical data is required to confirm the observations from the data in this study. Such strategies could include qPCR estimates of abundance of TM7 taxa in the soils as well as *in vitro* experiments involving manipulated soil parameters. Additionally, further exploration of the biological associations of the phylum would give clues to potential metabolic requirements through analysis of supernatant of co-occurring species that exist as pure cultures. However, the characteristics that define the preferred conditions of each class are likely to follow the observed correlations. Therefore, TM7-1 are endemic in moist soils that are acidic, rich with total iron, silicon, titanium and copper, and a mean grain size greater than 2 mm. This suggests they are biofilm-forming species and potentially have a facultative anaerobic metabolism. TM7-3 are probably oligotrophic heterotrophs, favoring dry soils that are alkaline with plentiful nutrient sources such as phosphate, iron, and sodium. In addition, the phylum appears to be tolerant of toxic mixtures such as diesel fuel, which lends to its ability to be so cosmopolitan. It is not clear whether these conditions directly give rise to or provide the nutrients for the growth of TM7 or whether they inhibit or promote the growth of other microorganisms providing TM7 with a niche to thrive.

Exploration of potential biological factors revealed that the TM7-3 class was the only one that responded to microbial community parameters. These responses were negative and show that the class favors a community of fewer members with an uneven community profile. It is likely that this is due to increased niche availability and less competition for resources. The biological relationships observed through the co-occurrence analysis provide an insight to the types of bacteria that members of the TM7 phylum are likely to associate with in a polar setting. Of particular interest was the relationship the two classes had with the *Gemmatimonadetes* phylum. This phylum is only recently characterized (2003) but members of this group are known to be poly-phosphate accumulating organisms first isolated in activated sludge (Zhang, 2003). This phenotype provides and interesting situation with the groups negative correlation to the TM7-3 class. One suggestion for this negative correlation for two groups that are positive associated with phosphates would be competitive inhibition, whereby both groups are competing for the phosphate availability in the soil (which compared to sludges is low). There are many correlations to candidate phyla for both the TM7-1 and TM7-3 class which since they are candidate groups little can be derived from these association as little is known about these phyla. The TM7-3 clade also has a negative association with the *Acidobacteria*, (while the TM7-1 class has a positive one) and since many members of this phylum are acidophiles, this strengthens the association the TM7-3 clade has for an alkaline environment as a preferred niche whereas TM7-1 members prefer acidic soils.

This study is an exploratory pilot in establishing a direction to pursue cultivation efforts for bacteria from the TM7 phylum. However, the large number of samples and span of locations increases the likelihood that these correlations are not merely coincidental though findings must be adopted with caution. There are a number of areas that could be explored to establish stronger links between the TM7 phylum's members and their nutritional requirements. One point of consideration is that the data presented here are from a 16S survey and the TM7 abundance data is relative to the depth of sequencing. A caveat associated with this is that while relative abundance may alter, absolute numbers might not. To obtain more confirmatory results, we would need to obtain data that incorporated biomass or absolute numbers of bacteria from the group. Quantitative PCR with primers that target the TM7 phylum, such as those developed by Ferrari et al. (2014), can be used to quantify numbers of TM7 within the soil more accurately. This may provide stronger links to the observations in this study. Expanding on this, experimental manipulations of microcosms constructed from the polar soils were TM7 bacteria are abundant would allow exploration of the relationships derived here. Addition of P, TPH and adjustment of the pH would tease out these dependencies to confirm is they are in fact controlling the prevalence of the TM7 phylum. These manipulations could be couple with modeling techniques such as structural equation modeling to go beyond correlation and explore causal relationships for the TM7 phylum. Further genome analysis is a direction that is also required to uncover the requirements of the TM7 phylum, by sequencing more members, in the same manner as shown by Albertsen et al. (2013), to obtain more of the biochemical pathways possessed by members of the phylum. As only a few members of a ubiquitous phylum have been sequenced, only the surface has really been scratched in exploring the genomes of TM7 bacteria.

Here we are exploring the relationships between the ecological parameters of various polar soils and the TM7 phylum to gain insight into the potential requirements of the group for future efforts to bring members of the phylum into pure culture. So far, there have been limited studies into this group of bacteria with the majority being genome sequencing attempts (with recent success by Albertsen et al., 2013) and *in situ* hybridization to identify TM7 presence in various environments (sludge, dental soil etc). So far no one has explored the environmental requirements of members of the group, least of all across both class of the phylum. Based on this analysis, the next steps forward for cultivating TM7 may include a biofilm environment, with low pH, no free iron and reduced oxygen content, which may stimulate the TM7-1 class and an alkaline environment with plentiful phosphate and bioavailable iron that also contains hydrocarbons may stimulate bacteria belonging to the class TM7-3. Such conditions would provide the necessary environment for TM7 bacteria grown to microcolonies using methods such as the soil substrate membrane system to be transferred to successful *in vitro* cultivation (Ferrari et al., 2008). The prospect of obtaining pure or even highly-enriched cultures from members of the TM7 phylum, would allow genome sequencing efforts to derive the metabolic pathways of the group and a provide a platform to gain empirical data from this group of the *Bacteria*.

### Conflict of interest statement

The authors declare that the research was conducted in the absence of any commercial or financial relationships that could be construed as a potential conflict of interest.
