# Supplementary material for: The ecological controls on the prevalence of candidate division TM7 in polar regions
Source: Front Microbiol. 2014 Jul 15;5:345. doi: 10.3389/fmicb.2014.00345 (PMC4097103; doi:10.3389/fmicb.2014.00345)
Supplement: Supplementary file 1 [file Presentation1.PDF]

Supplementary Information

Table S1: The location and quantity of samples from each region in this study.

Pole	Site	Number of Samples
North	Alexandra Fiord, NU	18
North	Spitzbergen Slijeringa, Norway	18
North	Spitzbergen Vestpynten, Norway	18
South	Mitchell Peninsula, East Antarctica	93
South	Casey Station, Eastern Antarctica	24
South	Robinsons Ridge, Eastern Antarctica	18
South	Herring Island, Eastern Antarctica	18
South	Browning Peninsula, Eastern Antarctica	18

Table S2: A list of the various parameters of each soil sample that were obtained and the method used along with the detection limit of the method if available. * For elemental analysis a Mehlich-3 extraction method was used except in those soils with a pH greater than 8 where a neutral 1M ammonium acetate extraction method was used.

**Chemical/Parameter**	**Extraction Detection method**	**Detection Limit**	**Transformation method for analysis**
PO_4	0.5M bicarbonate/Colwell-P method and colorimetric molybdenum-blue determination	19 mg/kg DMB	Untransformed
NH_4	2M Kcl/colourimetric indophenol	0.75 mg/kg DMB	log(value)
C	Combustion and NDIR gas analysis	0.03% w/w	log(value)
conductivity	distilled water suspension	-----	Untransformed
pH	distilled water suspension	-----	Untransformed
Gravel size	laser scatter	-----	Untransformed

Mean grain size	laser scatter	-----	Untransformed
Mud size	laser scatter	-----	Untransformed
Sand size	laser scatter	-----	Untransformed
Al	Mehlich-3 extraction * ICP-OES (CEC)	3.1 mg/kg DMB	Untransformed
B	Mehlich-3 extraction * ICP-OES (CEC)	0.2 mg/kg DMB	Untransformed
Ca	Mehlich-3 extraction * ICP-OES (CEC)	1.3 mg/kg DMB	Untransformed
Cu	Mehlich-3 extraction * ICP-OES (CEC)	0 mg/kg DMB	Untransformed
Fe	Mehlich-3 extraction * ICP-OES (CEC)	0.3 mg/kg DMB	Untransformed
K	Mehlich-3 extraction * ICP-OES (CEC)	1.9 mg/kg DMB	Untransformed
Mg	Mehlich-3 extraction * ICP-OES (CEC)	0.3 mg/kg DMB	Untransformed
Mn	Mehlich-3 extraction * ICP-OES (CEC)	0.2 mg/kg DMB	Untransformed
Na	Mehlich-3 extraction * ICP-OES (CEC)	2.6 mg/kg DMB	Untransformed
P	Mehlich-3 extraction * ICP-OES (CEC)	0.9 mg/kg DMB	Untransformed
S	Mehlich-3 extraction * ICP-OES (CEC)	0.8 mg/kg DMB	Untransformed
Zn	Mehlich-3 extraction * ICP-OES (CEC)	0.9 mg/kg DMB	Untransformed
N	Total Kjedahl digestion	17 mg/kg DMB	log(value)
P	Total Kjedahl digestion	15 mg/kg DMB	log(value)
NH_4	water extraction/colourimetric indophenol	0.55 mg/kg DMB	square root(value)
Cl	water extraction/ion chromatography	2.02 mg/kg DMB	log(value)
NO_2	water extraction/ion chromatography	0.15 mg/kg DMB	log(value)
NO_3	water extraction/ion chromatography	0.76 mg/kg DMB	log(value)
PO_4	water extraction/ion chromatography	0.15 mg/kg DMB	log(value)
SO_4	water extraction/ion chromatography	0.15 mg/kg DMB	log(value)
Al_2O_3	X-ray fluourescence	10 ppm	log(100-value)
CaO	X-ray fluourescence	8 ppm	log(value)
Fe_2O_3	X-ray fluourescence	5 ppm	square root(value)
K_2O	X-ray fluourescence	7 ppm	untransformed
MnO	X-ray fluourescence	3 ppm	log(value)
Na_2O	X-ray fluourescence	16 ppm	untransformed
P_2O_5	X-ray fluourescence	4 ppm	log(value)
SiO_2	X-ray fluourescence	6 ppm	log(100-value)
SO_3	X-ray fluourescence	6 ppm	log(value)
TiO_2	X-ray fluourescence	7 ppm	untransformed
Dry matter fraction	Gravimetric	-----	log(1-value)

Table S3 Pearson coefficients and p-values of correlations between chemical and biological parameters and abundance of classes of the TM7 candidate phylum. The correlations shown are those that yielded significant p values (<0.05).

Class	Predictor	R	p-value
TM7-1	Total iron	0.430	0.000
TM7-1	Total silicon	0.307	0.005
TM7-1	Copper	0.278	0.013
TM7-1	Aspect	0.263	0.017
TM7-1	Total titanium	0.260	0.018
TM7-1	Gravel	0.255	0.021
TM7-1	Mud	-0.238	0.031
TM7-1	Total sodium	-0.254	0.021
TM7-1	Dry matter fraction	-0.263	0.017
TM7-1	Phosphate	-0.303	0.006
TM7-1	Total aluminium	-0.342	0.002
TM7-3	Phosphate	0.517	0.007
TM7-3	Phosphorus	0.514	0.009
TM7-3	Extractable Iron	0.459	0.021
TM7-3	Total Phosphorus	0.457	0.019
TM7-3	Sodium	0.454	0.023
TM7-3	Sodium CECe	0.451	0.024
TM7-3	Total phosphorus	0.433	0.021
TM7-3	pH	0.415	0.035
TM7-3	TPH (C9 to C40)	0.408	0.031
TM7-3	Total sodium	0.389	0.040
TM7-3	Bacterial Evenness	-0.408	0.031
TM7-3	Bacterial Richness	-0.452	0.016
TM7-3	Total titanium	-0.457	0.014
TM7-3	Total iron	-0.479	0.010

Table S4 DistLM ranking of variables that influence the TM7 phylum. The variables listed in the table are ranked in order from top to bottom of most influential on the TM7 phylum, with the R squared values and proportion of total influence listed.

Variable	R squared	p-value	Proportion
Total phosphorus	0.1048	0.001	5.25E-02
TPH (C9-C40)	0.31178	0.001	2.89E-02
pH	0.13256	0.001	2.78E-02
Phosphate	0.17654	0.001	2.63E-02
Total titanium	0.22245	0.001	2.53E-02
Total silicon	0.19716	0.001	2.06E-02
Total iron	0.24274	0.001	2.03E-02
NO_3	0.15023	0.01	1.77E-02
Total sodium	0.2587	0.009	1.60E-02
Sodium CECe	0.39388	0.009	1.59E-02
Mud	0.32531	0.019	1.35E-02
P_2O_5	0.27178	0.051	1.31E-02
Gravel	0.3382	0.039	1.29E-02
Zn	0.36869	0.043	1.24E-02
Moisture	0.28289	0.12	1.11E-02

Figure S1 Presence or absence of bacteria from the candidate phylum TM7 within this dataset. Samples in this nMDS ordination have a blue sized circle based on abundance if TM7 bacteria are detected, samples without TM7 are shown by open circles. The ordination was calculated with bacterial OTU abundances

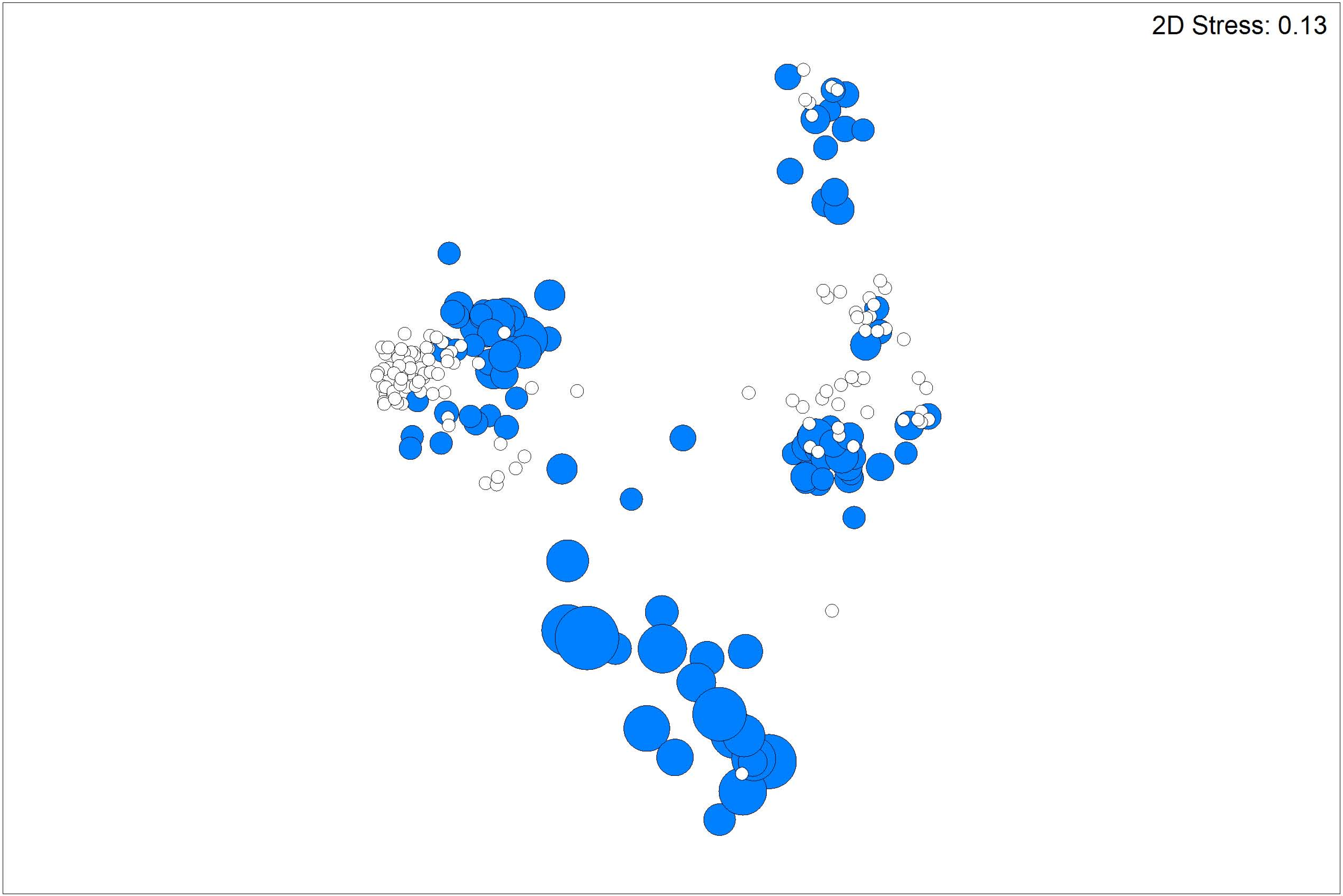
2D Stress: 0.13
